# Red meat intake is associated with early onset of rheumatoid arthritis: a cross-sectional study

**DOI:** 10.1038/s41598-021-85035-6

**Published:** 2021-03-11

**Authors:** Jiayang Jin, Jing Li, Yuzhou Gan, Jiajia Liu, Xiaozhen Zhao, Jiali Chen, Ruijun Zhang, Yan Zhong, Xiaomei Chen, Lijun Wu, Xiaohong Xiang, Yunshan Zhou, Jing He, Jianping Guo, Xu Liu, Zhanguo Li

**Affiliations:** 1 Department of Rheumatology and Immunology, Peking University People’s Hospital, Beijing, 100044 China; 2 Beijing Key Laboratory for Rheumatism Mechanism and Immune Diagnosis (BZ0135), Beijing, 100044 China; 3 Department of Rheumatology and Immunology, The People’s Hospital of Xin Jiang Uygur Autonomous Region, Urumqi, 830001 China; 4 Peking-Tsinghua Center for Life Sciences, Beijing, 100091 China

**Keywords:** Autoimmunity, Rheumatoid arthritis, Epidemiology

## Abstract

Accumulating evidence has implicated dietary factors as important risks for rheumatoid arthritis (RA) development, but analyses of the effects of red meat consumption on RA have yielded diverging results. The aim of this study was to explore the association between red meat and RA in a large-scale, cross-sectional study. From June to December 2016, a total of 733 patients were investigated, from which 707 participants were included in the analysis. These patients were divided into two groups according to their consumption of red meat (< 100 g/day; ≥ 100 g/day). The intake of red meat was assessed via physician-administered questionnaire. Generalized linear models were used to analyze relationships between the red meat intake and RA, adjusting for potential confounders including demographic, clinical, laboratory, and other dietary factors. Compared with low-intake red meat RA patients, high-intake red meat patients had an earlier onset age (*p* = 0.02) and had higher BMI (*p* = 0.003). The age at disease onset for the high-intake patients was 6.46 years earlier than for low-intake patients, after adjustment for demographic and other possible confounders (*β* = − 6.46, 95% CI − 9.77, − 3.15; *p* = 0.0001). Further, stratified analyses showed that this inverse association of red meat intake with RA onset age was especially evident in smokers and overweight patients (BMI ≥ 24 kg/m^2^). In conclusion**,** high-intake red meat is associated with early onset of RA, especially in smokers or overweight patients. The findings indicate that eating less red meat could be a recommendation given to patients at risk for RA development.

## Introduction

Rheumatoid arthritis (RA) is a systemic inflammatory disease of unknown etiology, characterized by symmetric arthritis and synovial inflammation^1^. The disease affects millions globally, and leads to progressive joint erosion and deformity^2^. RA is believed to be caused by a combination of genetic and environmental factors^3^. The genetic architecture of the disease has been well characterized through genome-wide analyses^4^. Environmental factors are considered to be important in triggering the onset of disease development in genetically susceptible individuals^3^. However, identifying triggers remains a challenge. Among environmental factors, smoking, obesity, and protein intake are considered to contribute to RA susceptibility^5^. Fish intake may decrease RA risk, potentially based on the anti-inflammatory properties of omega-3 polyunsaturated fatty acids (PUFAs)^6^.

Components of cooked red meat such as saturated fat, polycyclic aromatic hydrocarbons, and various preservatives can contribute to adverse health outcomes^7^.The association between red meat and RA is controversial. A study from 2000 used an ecologic approach and reported a positive correlation between the national prevalence of RA and per capita consumption of red meat (r^2^ 0.877, *p* < 0.001 for eight countries)^8^. Moreover, a study found that a high intake of meat was associated with an increased risk of inflammatory polyarthritis^9^. However, Benito-Garcia and colleagues reported that red meat, poultry, and fish were not associated with RA risk in a large female cohort^10^. Therefore, in order to investigate whether red meat was associated with early onset age of RA, we explored a large cohort of patients in our hospital and analyzed the relationship between red meat intake and the onset age of RA based on smoking status, BMI, and other environmental RA risk factors.

## Methods

### Study design and participants

**Figure 1:**
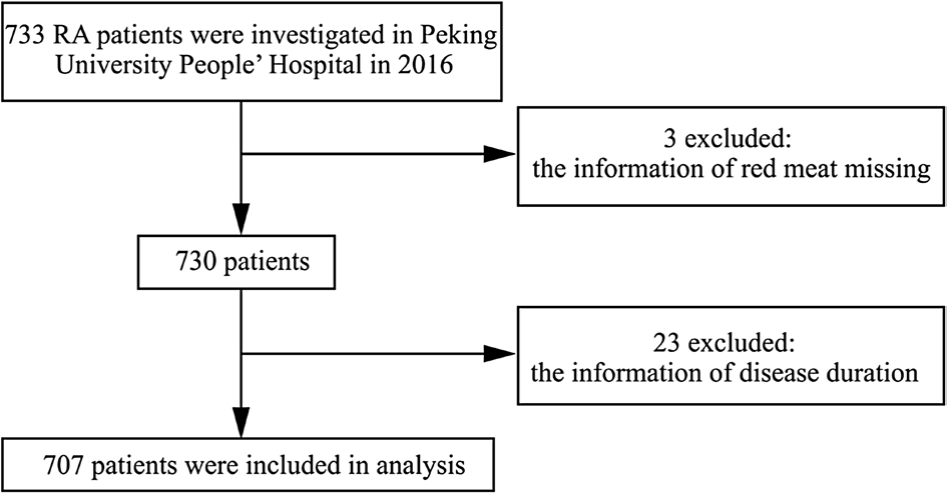
Patient flow chart. *RA* rheumatoid arthritis. A total of 733 RA patients were investigated in this study.

Between June and December 2016, a total of 733 participants were investigated from rheumatology clinics and wards of Peking University People’s Hospital (Fig. 1). RA patients were diagnosed in accordance with the American College of Rheumatology criteria^11^. A self-administered food frequency questionnaire was designed to measure socio-demographic, clinic characteristics, and dietary intakes during the years before RA symptom onset; all patients completed the questionnaire accompanied by a rheumatologist. The investigators were trained to administer the questionnaire and had all participated in medical research previously. The onset age of the disease was calculated by the patients age and time of onset of RA symptoms, not the time of RA diagnosis (Fig. 1). Note that among the 733 patients recruited into the study, 26 were excluded because they lacked adequately detailed information for red meat intake (3 patients) or for disease duration (23 patients). The study was approved by the Medical Ethics Committee of Peking University People’s Hospital. The number of the ethical committee approval were 2015PHB219-01 and the date is 2016-6-13. Informed written consent forms were obtained from all study participants. And the study was conducted in agreement with the Declaration of Helsinki and met the eligibility criteria according to the STROBE (Strengthening the Reporting of Observational Studies in Epidemiology).

### Dietary assessment

Dietary assessment on the food frequency questionnaire during the years before RA symptom onset included the frequency and the amount of dietary intake: the habitual intake of red meat (pork, beef, mutton, venison, rabbit) and white meat & sea food (fish, shrimp, chicken, duck, shellfish) were categorized as 0–100 g/day and ≥ 100 g/day. Sodium intake behavior was measured with a range from 1 to 3 (sodium: 1 ≤ 6 g/day, 2 = 6–12 g/day, 3 ≥ 12 g/day). Moreover, the assessment of different kinds of beverages included intake of milk, soybean milk, and yoghurt range from 1 to 3 [milk and soybean milk: 1 = No, 2 = 1–3 cups/week, 3 = 4 or more cups/week (200 ml/cup); yoghurt: 1 = No, 2 = 1–4 cups/week, 3 = 5 or more cups/week (50 ml/cup)]. The total amount of dietary intake per day or per week of a food item was calculated as the intake amount per serving of that food item × the frequency of dietary intake.

### Demographic and clinical assessment

Demographic and clinical data including as age, sex, nationality, Body Mass Index (BMI), alcohol drinking, smoking habits at the time of onset of RA symptoms, rheumatic disease family history (rheumatoid arthritis, systemic lupus erythematosus, Sjogren syndrome, primary biliary cirrhosis), disease duration, rheumatoid factor (RF), and anti–citrullinated protein antibody (ACPA) were collected from all patients. BMI (kg/m^2^) was calculated as weight (kg) divided by height squared (m^2^). Nationality was categorized as Han or minority. Smoking status at the time of onset of RA symptoms was categorized as non-smoker and smoker. Alcohol consumption was classified as non-drinker and drinker at the time of onset of RA symptoms.

### Statistical analysis

Descriptive statistics were used to describe the characteristics of the subjects using either the mean (standard deviation, SD) or median (interquartile range, IQR) for continuous variables, and frequency (percentage) for categorical variables. Mann–Whitney tests were used for non-normally distributed continuous variables. Chi-square tests were used to determine any statistical difference for proportions among two groups. Generalized linear models were applied to assess the association between red meat and onset age of RA. Interaction and stratified analyses were conducted according to sex, nationality, family history, BMI, smoking status, drinking status, levels of ACPA and RF, as well as intake of milk, soybean milk, yoghurt, white meat & sea food, and sodium. Each stratification adjusted for all the factors mentioned above, except the stratification factor itself. All of the analyses were performed with the statistical software packages R version 3.4.3 (http://www.R-project.org, The R Foundation) and EmpowerStats (http://www.empowerstats.com, X&Y Solutions, Inc., Boston, MA). A two-sided significance level of 0.05 was considered statistically significant.

## Results

### Characteristics of the patients

A total of 707 participants were included in the study of any associations between red meat and RA: 563 patients with low-intake of red meat (< 100 g/day) (79.63%) and 144 with high-intake (≥ 100 g/day) (20.37%). The characteristics of patients are outlined in Table 1. Compared with those in the low-intake of red meat group, the RA participants in the high-intake group were significantly younger (53.82 ± 13.53 vs 56.11 ± 13.36; *p* = 0.035), had earlier disease onset age (42.29 ± 14.08 vs 45.50 ± 14.76; *p* = 0.016), and were less likely to be female (63.19% vs 80.82%; *p* < 0.001), higher BMI (24.10 ± 3.76 vs 22.92 ± 3.46; *p* = 0.004).

**Table 1:** Characteristics of the RA patients by red meat consumption.

Variable	Total	< 100 g/day	≥ 100 g/day	*p*
N	707	563	144	
Age, mean (SD), years	55.64 ± 13.42	56.11 ± 13.36	53.82 ± 13.53	0.035
Sex, female, n (%)	546 (77.23%)	455 (80.82%)	91 (63.19%)	< 0.001
Nationality, Han, n (%)	655 (95.07%)	524 (95.62%)	131 (92.91%)	0.185
Family history, positive, n (%)	92 (14.56%)	71 (14.29%)	21 (15.56%)	0.711
BMI, mean (SD), kg/m^2^	23.16 ± 3.55	22.92 ± 3.46	24.10 ± 3.76	0.004
Drinking, n (%)	193 (28.26%)	144 (26.33%)	49 (36.03%)	0.024
Smoking, n (%)	241 (36.96%)	183 (35.26%)	58 (43.61%)	0.075
Disease duration, median (IQR), years	8.00 (3.00–16.00)	7.00 (3.00–16.00)	8.50 (4.00–16.00)	0.299
Onset age, median (IQR), years	44.84 ±14.67	45.50 ±14.76	42.29 ± 14.08	0.016
ACPA, positive, n (%)	554 (85.10%)	437 (84.53%)	117 (87.31%)	0.419
RF, positive, n (%)	433 (68.08%)	339 (67.53%)	94 (70.15%)	0.563
**Milk, n (%)**				0.974
No	244 (34.61%)	193 (34.40%)	51 (35.42%)	
< 800 ml/week	273 (38.72%)	218 (38.86%)	55 (38.19%)	
≥ 800 ml/week	188 (26.67%)	150 (26.74%)	38 (26.39%)	
**Soybean milk, n (%)**				0.449
No	315 (45.06%)	248 (44.52%)	67 (47.18%)	
< 800 ml/week	350 (50.07%)	284 (50.99%)	66 (46.48%)	
≥ 800 ml/week	34 (4.86%)	25 (4.49%)	9 (6.34%)	
**Yoghurt, n (%)**				0.471
No	237 (33.62%)	191 (34.05%)	46 (31.94%)	
< 200 ml/week	322 (45.67%)	250 (44.56%)	72 (50.00%)	
≥ 200 ml/week	146 (20.71%)	120 (21.39%)	26 (18.06%)	
**Sodium, n (%)**				0.035
< 6 g	293 (41.68%)	246 (43.69%)	47 (33.57%)	
6–12 g	219 (31.15%)	175 (31.08%)	44 (31.43%)	
> 12 g	191 (27.17%)	142 (25.22%)	49 (35.00%)	
**White meat and sea food, n (%)**				<0.001
< 100 g/day	610 (86.52%)	516 (91.81%)	94 (65.73%)	
≥ 100 g/day	95 (13.48%)	46 (8.19%)	49 (34.27%)	

*RA* rheumatoid arthritis, *BMI* Body Mass Index, *RF* rheumatoid factor, *ACPA* anti-citrullinated protein antibody.

The high-intake red meat RA participants were also more likely to have higher intake for sodium (*p* = 0.035), white meat and sea food (*p* < 0.001), and alcohol (*p* = 0.024); note of the other diet factors exhibit any statistically significant differences between the low- and high-intake red meat groups [including the intake of milk (*p* = 0.974), soybean milk (*p* = 0.449) and yoghurt (*p* = 0.471)]. Nor was there any significant difference between the two groups in terms of nationality, smoking status, family history of rheumatoid disease, disease duration, or ACPA and RF antibody levels.

### Association of red meat consumption with the age at RA onset

An analysis of association between intake of red meat and onset age of RA patients is presented in Table 2. In the univariate model, compared with patients of the low-intake of red meat group, the age at disease onset of the high-intake group was significantly earlier (*β* = − 3.20, 95% CI − 5.88, − 0.53, *p* = 0.0193) (Table 2). This association remained significant after adjustment for sex, BMI, family history, nationality, drinking, and smoking status (Model I) (*β* = − 5.59, 95% CI − 8.69, − 2.50, *p* = 0.0004). In the multivariable model which additionally adjusted for ACPA and RF (Model 2), the *β* for all RA was − 6.14 (95%CI − 9.20, − 3.08; *p* = 0.0001). After full adjustment for demographic characteristics, clinical features, and diet factors (Model 3), the age at disease onset in patients with high intake of red meat was 6.46 years earlier than that of low-intake (*β* = − 6.46, 95% CI − 9.77, − 3.15, *p* = 0.0001).

**Table 2:** Relationship between red meat with onset age of RA in different models.

	*β*	95% CI	*p*
**Red meat (< 100 g/day, reference group)**	*–*	*–*	*–*
Crude model^a^	− 3.20	− 5.88 to − 0.53	0.0193
Model 1^b^	− 5.59	− 8.69 to − 2.50	0.0004
Model 2^c^	− 6.14	− 9.20 to − 3.08	< 0.0001
Model 3^d^	− 6.46	− 9.77 to − 3.15	0.0001

*RA* rheumatoid arthritis.

^a^Crude model: we did not adjust other covariates.

^b^Model 1 adjusted for: drinking, smoking, sex; BMI; family history, nationality.

^c^Model 2 adjusted for: drinking, smoking, sex; BMI; family history, nationality, ACPA, RF.

^d^Model 3 adjusted for: drinking, smoking, sex; BMI; family history, nationality, ACPA, RF, white meat and sea food, sodium, milk, yoghurt, soybean milk.

### Subgroup analyses of factors influencing the association between red meat and the disease onset of RA

**Figure 2:**
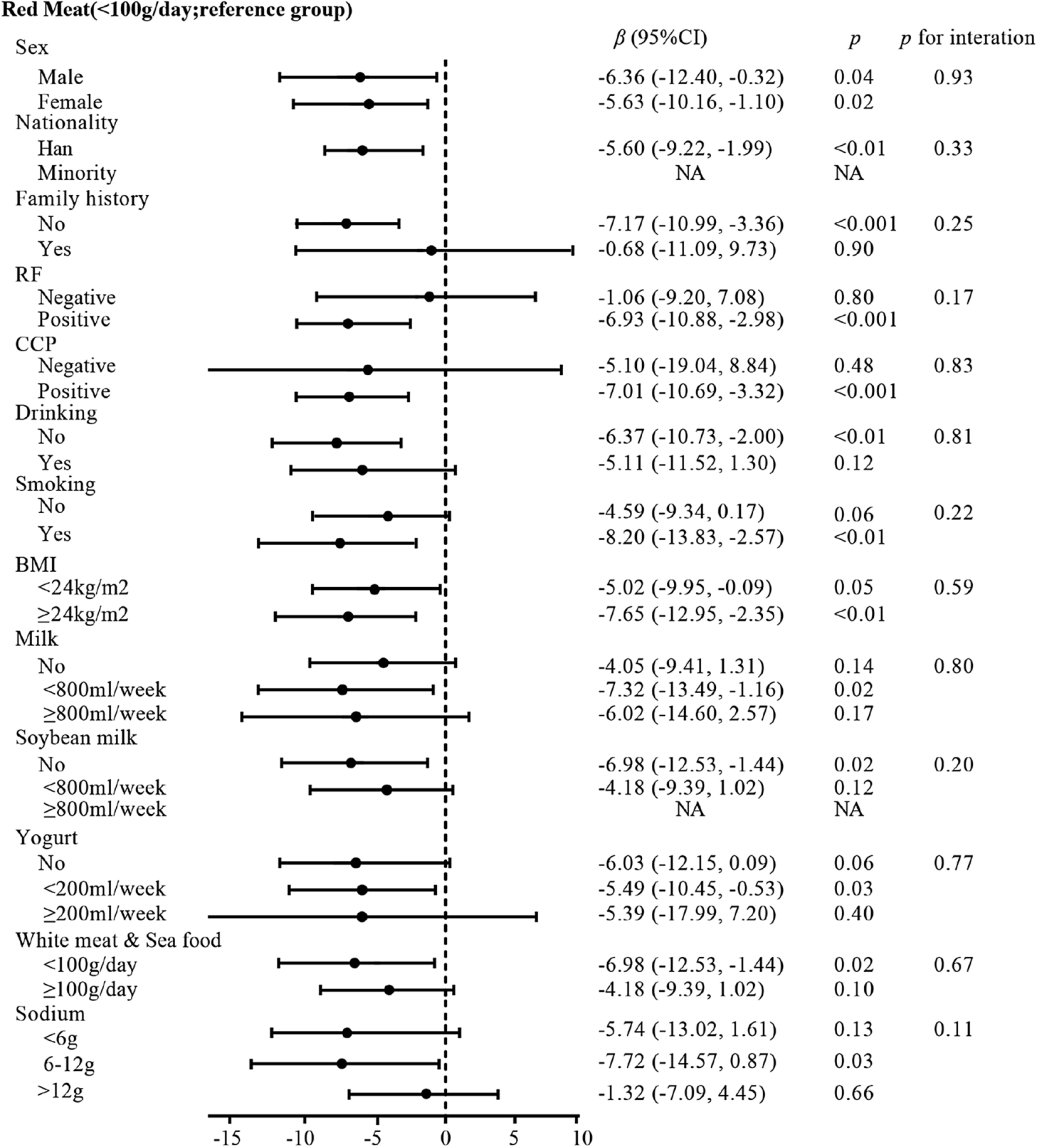
The association between red meat consumption and onset age of RA, stratified by the covariates. *BMI* Body Mass Index, *RF* rheumatoid factor, *ACPA* anti-citrullinated protein antibody. Each stratification adjusted for all the factors (sex, nationality, family history, RF, ACPA, drinking status, smoking status, BMI, as well as intake of milk, soybean milk, yoghurt, white meat and sea food, and sodium) except the stratification factor itself.

The effect of red meat consumption on the disease onset of RA patients stratified by the covariates are shown in Fig. 2. Each stratification was adjusted for all the factors (sex, nationality, family history, RF, ACPA, drinking status, smoking status, BMI, milk, soybean milk, yoghurt, white meat & sea food, and sodium) except the stratification factor itself. Stratified analysis showed that the association of higher red meat intake with earlier onset of RA was consistent, irrespective of sex, nationality, milk, yoghurt, sodium, or white meat and sea food intake level, smoking status, or alcohol consumption. The fact that these interactions between all covariables and the intake of red meat were not significant supports that the detected association of red meat intake and age of RA onset is stable among the different subgroupings (all *p* for interaction > 0.05).

In the smoking status subgroup, smokers with consuming red meat more than 100 g/day had advanced on-set age of RA (*β* = − 8.20, 95% CI − 13.83, − 2.57, *p* < 0.01) while the amount of red meat intake was not associated with non-smokers of RA (*β* = − 4.59, 95% CI − 9.34, 0.17, *p* = 0.06). Among overweight or obese participants, taking more than 100 g per day of red meat was associated with 7.65 years earlier onset of RA (*β* = − 7.65, 95% CI − 12.95, − 2.35, *p* < 0.01). However, among participants who were normal weight, the corresponding increased risk was not significant (*β* = − 5.02, 95% CI − 9.95, − 0.09, *p* = 0.05). There was no smoking-red meat (*p* for interaction = 0.22) or BMI-red meat (*p* for interaction = 0.59) interaction among the patients (Fig. 2).

## Discussion

In this cross-sectional study there was a significant inverse association between red meat intake and the onset age of RA, even when adjusting for sex, cigarette smoking, BMI, family history of rheumatic disease, and other related dietary factors. A previously reported nested case–control study from the UK showed that high level consumption of red meat is an independent risk factor for the development of inflammatory polyarthritis^9^. Our results for a Chinese RA patient cohort were similar to the UK study, although the typical diet composition between these two countries is different.

In contrast, there is another study from the USA which reported no RA risk association for consumption of red meat, poultry, or fish^10^. This discrepancy with the USA study can potentially be attributed to methodological differences and/or ethnic differences. Our criterion for red meat consumption was defined on a gram/day basis, whereas their questionnaire focused on consumption times/day. Hu et al.^12^ reported that among the individual components of the AHEI-2010 score, lower red meat intake was associated with decreased early-onset RA risk in American women^12^. In our study, male patients accounted for a larger proportion of the high-intake red meat consumption group (36.81% vs 19.18%, *p* < 0.001).

Our study provides data supporting a detailed analysis of the association of RA onset age and the level of red meat intake. Strengths of our study include the large number of participants and the ability to adjust for known and potential confounders. Sodium intake as a covariate was included, as high sodium consumption has been previously associated with an increased risk of RA, perhaps via a mechanism involving expansion of Th17 cells through activation of serum glucocorticoid kinase^13,14^. Our multivariable models were adjusted for 10 variables that are known or suspected confounders: this analysis revealed that the age at disease onset in high-intake red meat RA patients was 6.46 years earlier than for low-intake RA patients.

The primary advantages represented by the present are those afforded by its the detailed nature of the dietary inquiry and its stratified analysis. This finding would guide the clinicians to make accurate recommendation for the daily diet of RA patients. In the smoking subgroup, smoking patients of the high-intake red meat group had an earlier onset age, whereas non-smokers did not. And similar results were found for overweight and obese patients as compared to normal weight RA patients. As a clinician, we may advise individuals at risk for RA to eat less red meat, especially for those who were overweight and smoker.

A lot of evidence has shown that higher red meat consumption is associated with an increased risk of cardiovascular disease, colorectal cancer, and autoimmune diseases including rheumatoid arthritis (RA)^15–17^. A recent study of general mortality rates reported that increased red meat consumption is associated with a higher mortality risk women and men^18^. In a recent study, Christ et al.^19^ identified a functional role for NLRP3/IL-1β in the induction of innate immune memory in monocytes as triggered by western diets typified by high intake of red meat, and showed how this promotes atherosclerosis and inflammatory diseases^19^. It should be noted that cooked red meat contains not only saturated fat, polycyclic aromatic hydrocarbons, and iron, but can also be modified to enhance flavor or improve preservation through methods such as curing, smoking, salting, or even the addition of chemical preservatives^20,21^. Exposure to high temperatures can generate high levels of advanced glycation end-products in meat, which have been shown to increase oxidative and inflammatory processes^22^. The iron content in red meat has been linked to upregulate inflammatory mediators, such as IL-6, IL-8, IL-1β, and tumor necrosis factor alpha (TNF-a)^23^. Furthermore, it has also been demonstrated that there are gut microbiota-dependent breakdown metabolic processes for red meat that can promote inflammatory disease^24,25^.

Some limitations of the present study should be considered in light of the results. First, most subjects were recruited from the Han nationality, and further studies in national minority are needed. As reported, the age of RA onset associated with the degree of smoking and drinking, we would supplement these questions in the next study. Second, patient recall bias on the information of diet factors is another possible limitation especially since these were collected a mean of 11 years after the onset of RA symptoms. Third, combining white meat and seafood is a weakness of the data. Fourth, our study had a cross-sectional design, which did not enable us to draw any conclusions about causal relationship(s) between red meat and RA development. Therefore, prospective cohort studies are recommended.

## Conclusions

In conclusion, this study shows high-intake red meat is associated with early onset of RA. Furthermore, our findings emphasize the need to investigate the combination effect of red meat and smoking or overweight”.

## Data Availability

The data used to support the findings of this study are available from the corresponding author upon request.

## References

[1] Moller-Bisgaard S (2019). Effect of magnetic resonance imaging vs conventional treat-to-target strategies on disease activity remission and radiographic progression in rheumatoid arthritis: The IMAGINE-RA rando. JAMA.

[2] Assayag D, Lee JS, King TE (2014). Rheumatoid arthritis associated interstitial lung disease: A review. Medicina.

[3] McInnes IB, Schett G (2017). Pathogenetic insights from the treatment of rheumatoid arthritis. Lancet.

[4] McInnes IB, Schett G (2011). The pathogenesis of rheumatoid arthritis. N. Engl. J. Med..

[5] Liao KP, Alfredsson L, Karlson EW (2009). Environmental influences on risk for rheumatoid arthritis. Curr. Opin. Rheumatol..

[6] Sparks JA (2019). Association of fish intake and smoking with risk of rheumatoid arthritis and age of onset: A prospective cohort study. BMC Musculoskelet. Disord..

[7] Sieberts SK (2016). Crowdsourced assessment of common genetic contribution to predicting anti-TNF treatment response in rheumatoid arthritis. Nat. Commun..

[8] Grant WB (2000). The role of meat in the expression of rheumatoid arthritis. Br. J. Nutr..

[9] Pattison DJ (2004). Dietary risk factors for the development of inflammatory polyarthritis: Evidence for a role of high level of red meat consumption. Arthritis Rheum..

[10] Benito-Garcia E, Feskanich D, Hu FB, Mandl LA, Karlson EW (2007). Protein, iron, and meat consumption and risk for rheumatoid arthritis: A prospective cohort study. Arthritis Res. Ther..

[11] Aletaha D (2010). 2010 Rheumatoid arthritis classification criteria: An American College of Rheumatology/European League Against Rheumatism collaborative initiative. Arthritis Rheum..

[12] Hu Y (2017). Long-term dietary quality and risk of developing rheumatoid arthritis in women. Ann. Rheum. Dis..

[13] Sundstrom B, Johansson I, Rantapaa-Dahlqvist S (2015). Interaction between dietary sodium and smoking increases the risk for rheumatoid arthritis: Results from a nested case-control study. Rheumatology.

[14] Binger KJ (2015). High salt reduces the activation of IL-4- and IL-13-stimulated macrophages. J. Clin. Invest..

[15] Abbasi J (2019). TMAO and heart disease: The new red meat risk?. JAMA.

[16] Godfray HCJ (2018). Meat consumption, health, and the environment. Science.

[17] Clemente JC, Manasson J, Scher JU (2018). The role of the gut microbiome in systemic inflammatory disease. BMJ.

[18] Zheng Y (2019). Association of changes in red meat consumption with total and cause specific mortality among US women and men: Two prospective cohort studies. BMJ.

[19] Christ A (2018). Western diet triggers NLRP3-dependent innate immune reprogramming. Cell.

[20] Bouvard V (2015). Carcinogenicity of consumption of red and processed meat. Lancet Oncol..

[21] Wolk A (2017). Potential health hazards of eating red meat. J. Intern. Med..

[22] Clarke RE, Dordevic AL, Tan SM, Ryan L, Coughlan MT (2016). Dietary advanced glycation end products and risk factors for chronic disease: A systematic review of randomised controlled trials. Nutrients..

[23] Klaunig JE, Kamendulis LM (2004). The role of oxidative stress in carcinogenesis. Annu. Rev. Pharmacol. Toxicol..

[24] O'Neill LAJ, Zaslona Z (2018). Macrophages remember cheeseburgers and promote inflammation via NLRP3. Trends Mol. Med..

[25] Koeth RA (2013). Intestinal microbiota metabolism of l-carnitine, a nutrient in red meat, promotes atherosclerosis. Nat. Med..

